# Pathways to care and service preferences, and experiences of women who self-manage abortion through community pharmacies in two counties of Kenya

**DOI:** 10.1136/bmjgh-2025-021247

**Published:** 2026-03-27

**Authors:** Francis Obare, Steve Biko Sigu, Wilson Liambila, Beatrice Otieno, Lucy Nyamwaro, Kristen Shellenberg

**Affiliations:** 1 Population Council, Nairobi, Kenya; 2 Ipas Africa Alliance, Nairobi, Kenya; 3 Ipas USA, Chapel Hill, North Carolina, USA

**Keywords:** Health Services Accessibility, Kenya, Pregnancy

## Abstract

**Introduction:**

This study examines women’s pathways to medication abortion (MA) and post-MA contraception, including how they source information on the services, their choices and preferences regarding these services and their experiences obtaining the services from community pharmacies in two counties of Kenya.

**Methods:**

Data are from the baseline of a mixed-methods prospective intervention study conducted in two counties of Kenya (Nakuru and Kericho) between May 2024 and June 2025. We recruited women from 43 community pharmacies (27 in Nakuru and 16 in Kericho county). Data collection involved a quantitative survey and in-depth interviews with women.

**Results:**

A total of 524 women aged 17–48 years completed the quantitative survey while another 42 women aged 19–40 years (not in the quantitative survey) completed in-depth interviews at baseline (3–7 days following MA purchase). Most women relied on social networks (63% in Nakuru, 85% in Kericho) or pharmacies (35% in Nakuru, 56% in Kericho) for information on MA services. However, women themselves made the final decision to obtain MA. Women valued privacy, confidentiality and trust in pharmacy staff, with 94% of those in Nakuru and 88% of those in Kericho preferring the same pharmacy for post-MA contraceptive methods. Women generally preferred obtaining MA and post-MA contraceptive services separately to allow for the abortion process to be complete and to avoid potential adverse reactions from MA and contraception. They were also generally satisfied with the services they received from the pharmacy, mainly because of privacy, friendliness of the provider, quality of information provided, effectiveness of the medications provided and follow-up conducted by pharmacy staff.

**Conclusion:**

Community pharmacies are critical points of care for MA and post-MA contraception, providing privacy and convenience to underserved populations. Capacity-strengthening for pharmacists should focus on rights-based, comprehensive counselling to improve sexual and reproductive health outcomes.

**WHAT IS ALREADY KNOWN ON THE STUDY TOPIC**

- Women in low-resource settings face several barriers to accessing medication abortion (MA) information and services, including limited information on availability of services, stigma, legal restrictions around abortion and provider biases and limited capacity to offer comprehensive care.
- Understanding women’s agency to overcome these barriers is important for informing the design of context-specific interventions that account for the constraints within which they make decisions regarding their health.

**WHAT THIS STUDY ADDS**

- Women from low-resource settings have the agency to overcome barriers to accessing MA information and services, and value privacy, confidentiality and trust in pharmacy staff.
- Community pharmacies are critical points of care for MA and post-MA contraception, providing privacy and convenience to underserved populations.

**HOW THIS STUDY MIGHT AFFECT RESEARCH, PRACTICE OR POLICY**

- Community pharmacists require capacity strengthening in order to provide quality care to women seeking MA services in a manner that upholds users’ rights to choice and preferences.
- This requires in-service training for those already on the job as well as re-orienting the pre-service training curriculum to incorporate comprehensive sexual and reproductive healthcare.
- Given the limited method mix, there is a need to strengthen referral linkages between community pharmacies and public or private health facilities that offer contraceptive methods not available at pharmacies, including tracking the referrals to support continuity of care and women’s service experiences.

## Introduction

 Self-care biomedical technologies, including medication abortion (MA), provide critical options for women in low-resource settings to access services outside traditional health facilities. However, barriers such as stigma, socio-cultural constraints and legal restrictions limit access to accurate information and quality care.^1^,^4^ Community pharmacies, as accessible points of care, can bridge these gaps, offering privacy, convenience and continuity of care for sensitive sexual and reproductive health (SRH) needs.^5^,^10^ Evidence, for instance, shows that 41% of women in 36 low- and middle-income countries (LMICs) who use private sector services obtain contraception from pharmacies or drug shops.^11^ Community pharmacists may, however, be constrained in providing quality care by their attitudes towards sensitive SRH issues such as abortion, the nature of their training which focuses on drug composition and dispensing of medicines as opposed to counselling clients, their business-oriented nature which may prioritise financial returns over provision of quality care and the clientele they serve who may value privacy and quick services more than receiving comprehensive care.^7 12 13^

Literature, for instance, shows that pharmacy staff introduce informal ways of determining women’s access to MA information and services based on assessment of legal risks and business interests and may therefore perpetuate barriers to accessing accurate information and quality care, especially for women from low socioeconomic backgrounds.^14^ A review of the literature on self-managed medication abortion in LMICs concluded that there was limited research on how services meet the needs of underserved populations such as those in rural areas or from low socio-economic backgrounds.^5^ An important research question is therefore how women in such contexts navigate the barriers to accessing MA information and services, including post-MA contraception, from community pharmacies. This study examines women’s pathways to MA and post-MA contraception, including how they access information on the services, their choices and preferences regarding these services, and their experiences obtaining the services from community pharmacies in two counties of Kenya. MA pills are registered in Kenya for duodenal ulcer, gastric ulcer, treatment of incomplete abortion and miscarriage, treatment and prevention of postpartum haemorrhage, treatment of missed abortion in the first trimester, treatment of intrauterine fetal death and cervical ripening, and are available with a prescription through pharmacies or clinics. This study specifically addresses the following research questions with respect to access to MA and post-MA contraception through community pharmacies:

1. How do women obtain information on MA services and what are their experiences obtaining the services from community pharmacies in the contexts of barriers such as stigma, socio-cultural constraints, potential provider biases and legal restrictions?
2. What are women’s preferences regarding MA and post-MA services provided by community pharmacists and what are the implications of these preferences for pharmacy provision of the services?

## Methods

### Study design

The study—the Post-Medical Abortion Contraception project—was a mixed-methods intervention study that employed both prospective and cross-sectional designs with intervention and comparison groups. Ipas, a global non-governmental organisation that works to end preventable deaths and morbidity from unsafe abortion, led the overall implementation of the project, including the implementation of interventions. Ipas implemented the project in collaboration with the County Governments of Nakuru and Kericho, Population Council, Impact for Health Initiative (IHI) and Nivi. Population Council was responsible for conducting the evaluation activities, the County Governments provided an enabling policy environment for implementing the study, Nivi led the implementation of the digital solution while IHI led a market systems development process aimed at developing strategies for situating the project within the broader MA and post-MA contraceptive service market.

### Study setting and period

We conducted the study in two counties of Kenya (Nakuru and Kericho) between May 2024 and June 2025. We purposively identified the counties based on attributes that provide potential for expanding post-abortion contraception, including the presence of transport hubs, flower farms (Nakuru) or tea farms (Kericho) and university campuses that might be associated with adverse SRH outcomes such as high rates of unintended pregnancy and unsafe abortions.

### Study population

The study involved data collection among two groups of women: a prospective quantitative survey with women accessing MA from community pharmacies in both counties, and prospective qualitative interviews with another set of women (not involved in the quantitative survey) in Nakuru county. This paper focuses on the baseline quantitative survey and qualitative interviews with women.

### Inclusion/exclusion criteria

We included married women aged 15–49 years or unmarried women between ages 18 and 49 years who purchased MA pills to end a pregnancy from participating pharmacies. We also included eligible women who purchased MA pills from participating pharmacies and who granted verbal consent and excluded those who did not grant consent. We also excluded unmarried girls aged below 18 years who purchased MA pills from the pharmacies due to challenges of obtaining parental/guardian consent given the sensitive nature of abortion. We further excluded women who obtained MA pills through proxies such as male partners because of privacy and confidentiality challenges associated with going through proxies. These criteria were used for identifying participants for both quantitative and qualitative interviews.

### Participant recruitment

We recruited women from 43 community pharmacies across the two counties (27 in Nakuru and 16 in Kericho) using standardised scripts provided to pharmacy staff. The scripts included a brief description of the study, captured the phone numbers of those who were willing to participate in the study and included a question asking willing participants to provide a code that they could remember to be used to confirm that they were the right person when contacted later. Research assistants followed-up with willing participants to schedule interviews. For qualitative interviews, the target was for each pharmacy in Nakuru to recruit two participants (one below the age of 25 years and another aged 25 years or older) while the rest of the participants were to be recruited for the quantitative survey given the larger sample size required compared with the qualitative sample. Women self-selected into the study based on whether they were comfortable talking to the research team after purchasing MA pills. All baseline interviews with women occurred 3–7 days following MA purchase and were conducted in person. In addition, the study investigators conducted regular visits to participating pharmacies to address any challenges with the recruitment process.

### Study parameters

During baseline, women were asked detailed questions about how they came to learn about the services, their decision-making process and preferences and experiences obtaining MA and post-MA contraception from community pharmacies. Participants in the quantitative survey were specifically asked about sources of information on MA, reasons for choosing the pharmacy for MA services, decision-making around MA and contraception, preferences for provision of MA and post-MA contraception (including timing, bundling or separating the services), and their interactions with pharmacy staff, including any experiences of provider influence on post-MA contraceptive decisions or stigmatising behaviour. Qualitative interviews explored participants’ expectations when seeking services from community pharmacies vis-à-vis their experiences, preferences for obtaining MA and post-MA contraception and reasons for the stated preferences, as well as their views about services obtained from community pharmacies. Qualitative interviews lasted between 16 and 62 min.

### Data analysis

We used Stata V.14 software to analyse quantitative data. Analysis involved generation of descriptive statistics and comparing distributions of key indicators by study site given variations in the distribution of participants by background characteristics between sites. We used an exploratory inductive content analysis approach to analyse the qualitative data from interviews with women. This involved reading and re-reading the transcripts in Word and outlining emerging themes, with supporting excerpts focused on the broad themes of interest in this study: how women sourced information on MA services, their choices and preferences regarding MA and post-MA contraceptive services and their experiences obtaining the services from the pharmacy.

### Ethical considerations

Research assistants obtained verbal informed consent before conducting the baseline interviews. In particular, key issues highlighted during the consent process included purpose of the study, institutional affiliation of the study team, general description of the research, reason for selecting the participant, duration of participation in the research, anticipated risks and discomfort of participating in the research, anticipated benefits of participation, right to decline or withdraw from the research at any time without reprisal, measures to ensure confidentiality (including keeping separate the recruitment form that had personally identifying information from the data as well as using unique codes generated for the purpose of the study to identify the data), name and contact of study investigators and name and contact of the representative of the national institutional review board that approved the research. The interviews took place at the pharmacies where women purchased MA and were scheduled during times of low client volumes, with pharmacy staff either providing a separate room or temporarily creating space within their outlets to allow the interviews to be conducted in private. The study compensated women who participated in the study at the equivalent of US$4 for transport to and from the interview venue.

### Patient and public involvement statement

The recruitment process considered the sensitive nature of abortion and contraception. First, the study investigators conducted sensitisation workshops for staff from potential pharmacies in the two study sites to introduce the study. We purposively identified pharmacies invited for the sensitisation workshops based on their location in the study sites, whether they stocked and sold MA pills directly to women and whether they had reasonable volumes of MA clients (approximately 10 clients a month). The study investigators used these sensitisation workshops to review the recruitment script with pharmacy staff. Following the sensitisation workshops, the study investigators visited the pharmacists at their premises to determine if they were willing to allow their outlets to be included in the study. A total of 34 pharmacies in Nakuru and 18 in Kericho participated in the sensitisation workshops. Similar to women, pharmacy outlets self-selected into the study based on whether they were comfortable with supporting the recruitment of MA clients.

## Results

### Characteristics of participants

Out of 634 women (459 in Nakuru and 175 in Kericho) purchasing MA who were approached by pharmacy staff over a period of 8 months, 576 women (91%) agreed to be contacted by the research team and provided contact information that was recorded in the recruitment script (403 in Nakuru and 173 in Kericho), and 524 (91% of those who agreed to be contacted by the research team) were successfully interviewed at baseline (363 in Nakuru and 161 in Kericho). In-depth interviews were completed with another 49 women aged 19–40 years in Nakuru at baseline.

The distribution of participants in the quantitative survey by background characteristics varied by study site. 70% of those in Nakuru (N=363) were aged between 25 and 49 years while about two-thirds (63%; N=161) of those in Kericho were between the ages of 17–24 years (table 1). Similar patterns occurred with respect to distribution of participants by education level and marital status. About half (51%; N=363) of those in Nakuru had secondary-level education while close to two-thirds (63%; N=161) of those in Kericho had university or college level education. Two-thirds (66%; N=363) of those in Nakuru were married, living with a partner or had previously been married (separated, divorced or widowed) while 75% (N=161) of those in Kericho had never been married.

**Table 1:** Distribution of study participants by background characteristics

Characteristics	Nakuru (%)	Kericho (%)	Both sites (%)
Age group			
17–24	30.5	63.0	40.5
25–48	69.5	37.0	59.6
Marital status			
Never married	33.9	75.1	46.6
Married/living together	35.3	14.9	29.0
Formerly married*	30.9	9.9	24.4
Highest education level			
No schooling/primary	23.1	10.6	19.3
Secondary	51.2	26.1	43.5
College/university	25.6	63.4	37.2
Number of women	363	161	524

* Divorced, separated or widowed.

Among women in Nakuru who participated in the in-depth interviews at baseline (N=49), 55% were aged 25 years or older while 39% had never married, 35% were formerly married and 26% were married or living with a man at the time of interview.

### MA and post-MA contraceptive information pathways

**Figure 1:**
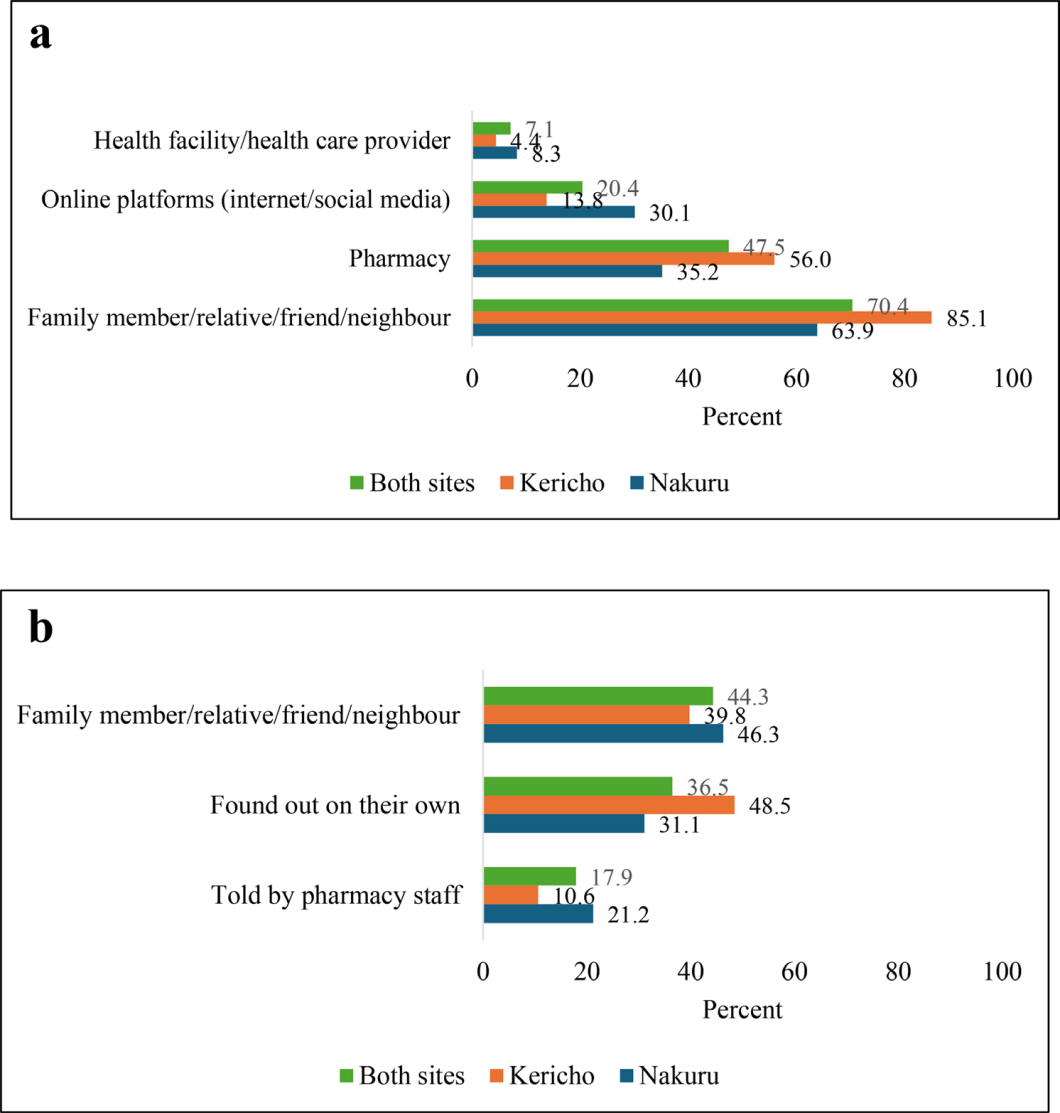
(**a–b**) Most common sources of information on medication abortion pills and provider. (**a**) Note: Question allowed multiple responses; number of women: Nakuru=363; Kericho=161. (**b**) Number of women: Nakuru=363; Kericho=161.

A majority of women relied on social networks such as family members, relatives, friends and neighbours (63% in Nakuru, N=363; 85% in Kericho, N=161) or pharmacies (35% in Nakuru, N=363; 56% in Kericho, N=161) for information on MA services; figure 1a). As a participant in the qualitative interviews narrated, “Okay even for abortion, she [friend] is the one who had informed me I can get the services,” (never-married 20-year-old participant). Qualitative data further showed that women who obtained information on MA services from pharmacies were largely those who walked from pharmacy to pharmacy to inquire about services (“I asked around as I hopped from pharmacy to pharmacy, the first one, second one and this was the third one. Most of them were refusing, but I can say I really walked trying to get help”—formerly married 25-year-old participant) or were already acquainted with the pharmacy staff (“I am a regular customer here”—formerly married 30-year-old participant). Results from the quantitative survey further showed that online platforms such as internet and social media were the third most common source of information on MA (mentioned by 30% of women in Nakuru, N=363, and 13% of those in Kericho, N=161; figure 1a).

Family members, relatives, friends and neighbours were also one of the most common sources of information on the pharmacy where women obtained MA (cited by 46% of those in Nakuru, N=363, and 40% of those in Kericho, N=161; figure 1b). As a participant in the qualitative interviews explained, “Initially, there is a friend who told me to come and talk to [name of pharmacy staff]. She told me she had also received help from the chemist sometime back and encouraged me to go and talk to the provider”, (married 40-year-old participant). A substantial proportion also found out about the pharmacy on their own (31% in Nakuru, N=363; and 49% in Kericho, N=161) or were told by pharmacy staff (21% in Nakuru, N=363; and 11% in Kericho, N=161; figure 1b). A participant in the qualitative interviews, for instance, reported that, “She [pharmacy staff] is the one who told me [about MA]. She told me if I wanted or if I knew a client who needed such services then I could refer to her”, (formerly married 36-year-old participant).

### Reasons for choice of pharmacy

The most common reasons for the choice of the specific pharmacy where women obtained MA included trust or acquaintance with the pharmacy or pharmacy staff (mentioned by 58% of women in Nakuru, N=363; and 50% of those in Kericho, N=161), discrete, private or confidential nature of services (47% in Nakuru, N=363; and 42% in Kericho, N=161), recommendation by someone (45% in Nakuru, N=363; and 41% in Kericho, N=161) and ease of access (41% in Nakuru, N=363; and 47% in Kericho, N=161; table 2). These considerations were also consistent with sentiments expressed in the qualitative interviews, for example: “I prefer him [pharmacy staff] because perhaps he has been my go-to provider”, (36-year-old formerly married participant), “This was the nearest pharmacy and we know each other”, (never married 24-year-old participant) or “What impressed me most about this place is the kind of privacy around here”, (formerly married 33-year-old participant).

**Table 2:** Reasons for choice of pharmacy and decision-making regarding medication abortion and contraception

Indicator	Nakuru (%)	Kericho (%)	Both sites(%)
Reason for choice of pharmacy*			
Trust/acquaintance with provider/place	57.9	49.7	55.3
Discreteness/privacy/confidentiality	46.6	42.2	45.2
Recommended by someone	44.9	41.6	43.9
Ease of access	40.5	47.2	42.6
Inexpensive	17.9	16.2	17.4
It was the only place known to respondent	10.2	5.0	8.6
Efficient/little time required to get service	3.0	6.2	4.0
Non-invasive/does not access a lot of details	3.9	2.5	3.4
Referral by ask Nivi†	1.1	0.0	0.8
Final decision to obtain MA pills			
Woman/self	84.9	58.4	76.7
Husband/partner	5.2	9.9	6.7
Joint decision with husband/partner	9.1	30.4	15.7
Pharmacy staff	0.3	0.0	0.2
Family member/relative/friend/neighbour	0.6	1.2	0.8
Usual decision regarding contraception			
Woman/self	82.1	48.5	71.8
Husband/partner	3.3	11.8	5.9
Joint decision with husband/partner	14.3	39.8	22.1
Other	0.3	0.0	0.2
Final decision regarding contraception‡			
Woman/self	82.4	56.5	74.4
Husband/partner	11.9	21.7	14.9
Joint decision with husband/partner	4.1	21.7	9.5
Other	1.7	0.0	1.1
Number of women	363	161	524

* Question allowed multiple responses.

† Digital intervention implemented in Nakuru county.

‡ In cases of disagreement.

MA, medication abortion.

### Decision-making and autonomy

Although family members, relatives, friends or neighbours were the most common sources of information on MA or MA provider, they did not play a major role in women’s final decision to use MA. Rather, for the most part, women themselves made the final decision to use MA (85% of those in Nakuru, N=363; and 58% of those in Kericho, N=161), with a substantial proportion of women in Kericho compared with Nakuru reporting partner involvement in the final decision-making (40%, N=363; and 14%, N=161, respectively; table 2). Similar patterns occurred with respect to regular or final decisions around contraception. This was consistent with sentiments expressed in qualitative interviews, that although most women relied on friends or relatives—some of whom had experienced an abortion—on information on availability of abortion pills and where to obtain them, the final decision on getting an abortion rested with themselves. For instance, “…my friend told me about this doctor [pharmacy staff], and I decided to make a visit and learn more about it”, (formerly married 33-year-old), or “I sat down and thought saying she [pharmacy staff] is the one who conducted the test … whom will I approach again for help? Then I just decided to tell her and asked her whether she has medications that can help me”, (married 29-year-old participant).

Insights from qualitative interviews further showed that for most women, the path to MA care was full of nervousness, while the decision to take up post-MA contraception for those who took up a method was not. Women reported being nervous about how they would be received at the outlets (“At first, I was nervous and it took me a lot of guts to tell her [pharmacy staff] what was going on and why I want to do this”—formerly married 30-year-old participant or “I was expecting she [pharmacy staff] would berate me, scold me”—never married 21-year-old participant), anxious about where or how to start the conversation with the pharmacy staff (“So at the time I was coming I was anxious on where to start, how I’ll start, the kind of person I’ll encounter”—formerly married 38-year-old participant), worried or afraid of both the health and legal consequences of their actions (“I was thinking I may die”—never married 20-year-old participant, or “I thought I would be arrested”—never married 22-year-old participant), or confused (“I was confused to the extent that I had no expectations”—never married 23-year-old participant). However, the transition to post-MA contraception was straightforward for those who took up a method because of the desire to prevent another unintended pregnancy. As one participant explained, “I think it [post-abortion contraception] is important because you have plans. After an abortion, what is next? After an abortion, the next thing I am doing is I’m finishing up with reactions from my body then I am getting a method so that … even if I have sex with my partner, I’ll be totally safe”, (formerly married 26-year-old participant).

### Preferences for MA and Post-MA contraception

Most women preferred obtaining post-MA contraceptive information after knowing that the abortion is complete (60% of those in Nakuru, N=363; and 64% of those in Kericho, N=161) although a substantial proportion preferred getting such information when purchasing MA (32% in Nakuru, N=363; and 34% in Kericho, N=161). The patterns were also consistent with sentiments expressed in qualitative interviews, with most women preferring obtaining MA and post-MA contraception separately for reasons of allowing for the abortion process to be complete and avoiding any adverse reactions from using two hormonal products at the same time. As some participants reported, “I prefer having the MA process then come for the pills later to avoid any negative reaction afterwards”, (married 25-year-old participant) or “After getting the first, you are still under tension and you don’t know what will happen to you so after finishing with the first, your mind is settled and you can easily understand what you are being told”, (married 26-year-old participant). For participants who preferred getting contraception the same day, the main reason was the realisation of quick return to fertility following an abortion (“…you shouldn’t wait … The man can come by and you may find yourself pregnant again”,—married 22-year-old participant).

A majority of women in the quantitative survey preferred getting post-MA contraceptive information from the same pharmacy where they purchased MA (94% in Nakuru, N=363; and 54% in Kericho, N=161), with about a third (32%, N=161) of those in Kericho also preferring social media (WhatsApp or chatbot). Women valued privacy, confidentiality and trust in pharmacy staff, with 94% (N=363) of those in Nakuru and 85% (N=161) of those in Kericho preferring the same pharmacy for post-MA contraceptive method. A substantial proportion of those who used contraception prior to the index pregnancy that resulted in abortion and preferred the pharmacy as a source of post-MA contraception did not obtain their prior method from a pharmacy (40% in Nakuru, N=363; and 45% in Kericho, N=161), which was a shift in preference in favour of the pharmacy as a source of contraception following MA.

Qualitative interviews suggested that the shift in preference in favour of the pharmacy as a source of post-MA contraception was partly driven by a desire for continuity of care and to ensure confidentiality around the service (MA) that created the need for prevention of another unintended pregnancy. As some participants reported, “She [pharmacy staff] knows my story and understands everything about me”, (married 22-year-old participant) or “…the pharmacy where you obtained the abortion because they will understand you and how you got pregnant”, (married 26-year-old participant).

### Experiences with pharmacy providers

Results from a quantitative survey among women showed that, consistent with preferences for timing of post-MA contraception, a higher proportion obtained a method later compared with the proportion that obtained a method the same day they purchased MA. In particular, 51% (N=363) of women in Nakuru and 7% (N=161) of those in Kericho obtained a method two or more days following MA purchase, while 15% (N=363) of those in Nakuru and 2% (N=161) of those in Kericho obtained a method the same day they purchased MA. About one-third (34%, N=363) of women in Nakuru and 91% (N=161) of those in Kericho did not obtain a contraceptive method immediately following MA. Results further showed that pharmacy staff were better at performing certain post-MA contraceptive counselling tasks than others (table 3), with those in Nakuru doing better in most of the indicators compared with those in Kericho.

**Table 3:** Indicators of post-medication abortion contraceptive counselling by pharmacy staff at baseline

Indicator	Nakuru, (%)	Kericho, (%)	Both sites, (%)
Pharmacy staff asked questions to determine whether respondent was pregnant or not	98.7	93.3	98.4
Pharmacy staff gave information on available contraceptive methods	98.3	86.7	97.6
Pharmacy staff gave information on advantages and disadvantages of available contraceptive methods	97.1	86.7	96.4
Pharmacy staff gave respondent a chance to select preferred method	99.2	100.0	99.2
Pharmacy staff talked about when respondent is likely to get pregnant after ending a pregnancy	93.3	66.7	91.7
Pharmacy staff informed respondent to return to pharmacy in case of challenges when using method	99.6	100.0	99.6
Pharmacy staff gave respondent a card indicating the date on when to return to pharmacy	92.8	73.3	91.7
Pharmacy staff referred respondent elsewhere for preferred method	44.3	6.7	42.1
Pharmacy staff used written materials when giving information on contraceptive methods	83.1	46.7	81.0
Pharmacy staff used materials with pictures when giving information on contraceptive methods	83.1	53.3	81.4
Pharmacy staff said they would follow-up to find out how respondent is doing when using the method	100.0	73.3	98.4
Pharmacy staff asked whether respondent understood any information given	100.0	93.3	99.6
Pharmacy staff gave respondent an opportunity to ask questions	99.2	93.3	98.8
Number of women	237	15	252

Qualitative interviews with women in Nakuru county showed that they were generally satisfied with the services they received from the pharmacy, which was attributable to the friendliness of the providers (“I didn’t even know the pharmacist and the services were good … very friendly”,—married 37-year-old participant), quality of information provided (“All the information that I was given was effective and he is very knowledgeable”,—married 37-year-old participant), effectiveness of the medications provided (“… she [pharmacy staff] gave me the pills that helped me”,—Never married 21-year-old participant) and follow-up of clients (“She [pharmacy staff] used to contact me every day, asking me how I was doing. Even coming to my home to check up on me if I am doing well and advising me as in giving me information on what to do and what I am not supposed to do in the process”,—never married 22-year-old participant).

## Discussion and implications

### Programmatic implications

Consistent with existing research,^15^ the majority of women relied on social networks for information on availability of MA or source of care. Such information may not always be accurate; for instance, the fears around adverse reactions between MA and contraception expressed by some participants in the study and which is not supported by scientific evidence. This suggests a need for initiatives aimed at reaching women and their social networks with correct information on SRH self-care technologies, including MA. Community pharmacies play a pivotal role in bridging gaps in access to MA and post-MA contraception, particularly for underserved populations. Participants in this study particularly appreciated the quality of information they obtained from pharmacy staff, highlighting the potential for pharmacies to serve as trusted sites for information about medication abortion and contraception. Strengthening pharmacists’ capacity to provide comprehensive sexual and reproductive health counselling is therefore essential. Programmes or distributors of self-care technologies for SRH, such as MA, should therefore invest in strengthening the capacity of community pharmacists—who are often left out of regular on-the-job training provided to public sector healthcare workers—in comprehensive SRH counselling.

Women preferred community pharmacies for post-MA contraceptive methods, including those who had not previously obtained a method from these sources, for reasons of ensuring continuity of care and confidentiality of services given the MA entry point. However, further analysis of qualitative data showed that contraceptive method mix at pharmacies was limited to condoms, pills and injectables, with a few pharmacy staff informing clients about implants or intrauterine devices which required referrals. This suggests a need for strengthening the capacity of community pharmacists to provide referrals for methods they do not stock or lack the capacity to provide such as long-acting reversible contraceptives. This requires strengthening referral linkages between community pharmacies and public or private health facilities that offer contraceptive methods not available at pharmacies, including tracking the referrals to support continuity of care and women’s service experiences.

### System-level implications

At the systems level, a sustainable approach to strengthening the capacity of community pharmacists in comprehensive SRH counselling requires incorporating these aspects into their pre-service training curriculum. Given the business-oriented nature of community pharmacists, the training should incorporate rights-based principles to ensure that they provide services in a non-coercive manner. This is particularly important in the context of national and international guidelines that consider post-abortion contraception an integral component of comprehensive abortion care.^16^ In particular, strategies for improving uptake of contraception following abortion, such as supporting women to leave the health facility with a method, or in the case of MA, providing the pills together with a contraceptive method—referred to as ‘bundling’—remain contentious. On the one hand, such strategies reduce the risk of repeat unintended pregnancy following an abortion by ensuring that women take up a method almost immediately.^17^,^19^ On the other hand, the strategies may limit women’s rights to choice of whether to take up a method or not, which method to use and when to initiate method use.^20^ Training in rights-based health service provision can enable community pharmacists to support women to make informed decisions regarding their health.

### Study limitations

The study’s findings may be influenced by certain limitations. First, participating pharmacies and women were self-selected into the study based on their self-assessed level of comfort. It could be that pharmacies and individual women who declined to participate in the study were different in certain aspects from those who participated. However, given the small number of pharmacies and individual women who declined participation, it is unlikely that their experiences may significantly bias the study’s findings. Nonetheless, the self-selected nature of the sample could partially explain some of the differences between study sites. Second, the findings may reflect the views and experiences of women accessing MA services in the settings where the study was conducted and not those accessing similar services in other settings or those who use unsafe means to terminate pregnancies. Similarly, their views and experiences may not reflect those of unmarried girls under the age of 18 years who were excluded from the study due to ethical considerations around obtaining parental or caregiver consent. Third, findings are based on women’s self-reports and not objective measurement, and may therefore be influenced by desirability bias, especially if women anticipated some benefit from participation such as accessing care or financial gain.

## Conclusion

Self-care technologies remain underused and require support to ensure correct use for improved health outcomes. Community pharmacies are critical points of care for MA and post-MA contraception, providing privacy and convenience to underserved populations. Capacity-strengthening for pharmacists should focus on rights-based, comprehensive counselling to improve sexual and reproductive health outcomes.

## Supplementary material

10.1136/bmjgh-2025-021247 online supplemental file 1

## Data Availability

Data are available upon reasonable request.
