# Supplementary material for: Pathways to care and service preferences, and experiences of women who self-manage abortion through community pharmacies in two counties of Kenya
Source: BMJ Glob Health. 2026 Mar 27;10(Suppl 6):e021247. doi: 10.1136/bmjgh-2025-021247 (PMC13034278; doi:10.1136/bmjgh-2025-021247)
Supplement: online supplemental file 1 [file bmjgh-10-Suppl_6-s001.docx]

**BMJ Global Health Author Reflexivity Statement**

**Author Reflexivity Statement – Pathways to care and service preferences, and experiences of women who self-manage abortion through community pharmacies in two counties of Kenya**

| **Study conceptualisation** | |
| --- | --- |
| 1. How does this study address local research and policy priorities? | Post-abortion care (PAC) is a critical component of maternal health services in Kenya. The Kenya Ministry of Health guidelines on PAC emphasize training health care providers on treating complications, infection prevention, pain management, counselling and provision of post-abortion contraception. The study therefore addressed the national policy priorities of improving post-abortion contraception. |
| 1. How were local researchers involved in study design? | Local researchers from Population Council- Kenya (Francis Obare and Wilson Liambila) and program implementers from Ipas Africa Alliance (Steve Biko, Beatrice Otieno and Lucy Nyamwaro), also based in Kenya led the conceptualization, design and implementation of the study and the interventions, with technical support from Population Council, Inc (USA) and Ipas USA. All local researchers and program implementers are Kenyan citizens. |
| **Research management** | |
| 1. How has funding been used to support the local research team(s)? | The funding wholly supported local research and intervention implementation. Technical support provided by Population Council, Inc. and Ipas USA was covered under indirect costs. |
| **Data acquisition and analysis** | |
| 1. How are research staff who conducted data collection acknowledged? | Research and program staff who implemented the study are the authors of the paper. |
| 1. How have members of the research partnership been provided with access to study data? | Study data were shared with the members of research team through a dedicated SharePoint only accessible to members of the team. |
| 1. How were data used to develop analytical skills within the partnership? | All members of the research team reviewed the quality and completeness of the data collected, and provided insights on potential analysis during bi-weekly team calls. Team members also led the development of abstracts that were submitted to various conferences. |
| **Data interpretation** | |
| 1. How have research partners collaborated in interpreting study data? | All authors reviewed the study findings for substantial intellectual content. This was achieved not only through reviewing the paper, but also through reviewing presentations highlighting the study findings that were made to various in-country stakeholders, and at various conferences. |
| **Drafting and revising for intellectual content** | |
| 1. How were research partners supported to develop writing skills? | Team members took lead in developing abstracts that were presented at various conferences. A number of paper topics have also been identified and assigned to various team members to take lead on drafting full manuscripts. |
| 1. How will research products be shared to address local needs? | Study findings have already been shared with pharmacies that participated in the research, policy makers at national and county (Nakuru and Kericho) levels, and program implementers working on the sexual and reproductive health and rights space in Kenya through a series of dissemination meetings in order to inform strategies to improve post-medication abortion contraceptive service delivery through community pharmacies in the country. |
| **Authorship** | |
| 1. How is the leadership, contribution and ownership of this work by LMIC researchers recognised within the authorship? | All researchers and program implementers that led the implementation of the study and interventions are citizens of and are based in a low- and middle-income country (Kenya). |
| 1. How have early career researchers across the partnership been included within the authorship team? | The authorship team comprised three early career researchers (Steve Biko, Beatrice Otieno and Lucy Nyamwaro) |
| 1. How has gender balance been addressed within the authorship? | The authorship team comprises an equal number of female and male researchers, which by default, reflects the team that conceptualized and implemented the study. |
| **Training** | |
| 1. How has the project contributed to training of LMIC researchers? | Whereas there was no deliberate effort to train LMIC researchers, the early career researchers in the team gained hands-on experience in implementation research through their involvement in the day-to-day activities of the project. |
| **Infrastructure** | |
| 1. How has the project contributed to improvements in local infrastructure? | As part of the interventions, pharmacies participating in the project were provided with contraceptive seedstocks as well as training in commodity management and forecasting, which ensured that contraceptive methods were available to clients seeking services from the outlets even when there were stock-outs of commodities. Pharmacy staff were also trained on contraceptive counselling, which ensured improvements in the way they serve clients. |
| **Governance** | |
| 1. What safeguarding procedures were used to protect local study participants and researchers? | The study investigators obtained relevant ethical approvals (international and national) to ensure that the study conformed to standards of human subject protection. All women granted verbal informed consent before participating in the study; the study requested for a waiver of written consent from the ethical review boards given that it could be a source of identification of participants. Participants provided codes which research assistants used to identify them when contacted through phone to schedule interviews in order to ensure that the conversation did not get to individuals other than the participants. Interviews were conducted at the pharmacy where women purchased medication during times of low client volumes, with pharmacy staff either providing a separate room or temporarily creating space within their outlets to allow the interviews to be conducted in private. |
